# Long-term Effects of high-doSe pitavaStatin on Diabetogenicity in comparison with atorvastatin in patients with Metabolic syndrome (LESS-DM): study protocol for a randomized controlled trial

**DOI:** 10.1186/s13063-017-2229-4

**Published:** 2017-10-27

**Authors:** Jun-Bean Park, Ji-Hyun Jung, Yeonyee E. Yoon, Hack-Lyong Kim, Seung-Pyo Lee, Hyung-Kwan Kim, Yong-Jin Kim, Goo-Yeong Cho, Dae-Won Sohn

**Affiliations:** 1 Division of Cardiology, Seoul National University College of Medicine, Seoul, South Korea; 2 Cardiovascular Center, Seoul National University Hospital, 101 Daehak-ro, Jongro-gu, Seoul, 110-744 South Korea; 3 Cardiovascular Center, Seoul National University Bundang Hospital, Seongnam, South Korea; 4 Cardiovascular Center, SNU-SMG Boramae Medical Center, Seoul, South Korea; 5 Department of Internal Medicine, Seoul National University College of Medicine, 101 Daehak-ro, Jongno-gu, Seoul, 03080 South Korea

**Keywords:** Statin, Glucose metabolism, Adiponectin, Carotid elasticity, Cardiac function, Metabolic syndrome

## Abstract

**Background:**

The diabetogenic action of statins remains a concern, particularly in patients at high risk for diabetes receiving intensive statin therapy. Despite the risk of diabetes with statin use being considered a potential class effect, recent studies have suggested that pitavastatin exerts neutral or favorable effects on diabetogenicity. However, no randomized trial has compared the long-term effects of pitavastatin with those of other statins on glycemic control in populations at high risk for diabetes. Hence, we aim to assess the long-term effects of pitavastatin in comparison with atorvastatin on glucose metabolism in patients with metabolic syndrome (MetS).

**Methods/design:**

The Long-term Effects of high-doSe pitavaStatin on Diabetogenicity in comparison with atorvastatin in patients with Metabolic syndrome (LESS-DM) trial is a prospective, randomized, open-label, active control clinical trial of patients with MetS. We plan to randomize 500 patients with MetS (1:1) to receive high-dose pitavastatin (4 mg) or atorvastatin (20 mg) daily for 24 months. The primary endpoint will be the change in hemoglobin A1c after statin treatment. Secondary endpoints will include the following: (1) changes in biochemical markers, including insulin, C-peptide, homeostasis model assessment of insulin resistance and insulin secretion, and adiponectin; (2) changes in imaging parameters, including carotid elasticity metrics and indices of cardiac function; and (3) the incidence of clinical events, including new-onset diabetes and cardiovascular disease.

**Discussion:**

In this trial, we will explore whether pitavastatin 4 mg does not disturb glucose metabolism in patients with MetS. It will also provide mechanistic information on statin type-dependent diabetogenic effects and surrogate data regarding vascular and cardiac changes achieved by intensive statin therapy.

**Trial registration:**

ClinicalTrials.gov, NCT02940366. Registered on 19 October 2016.

**Electronic supplementary material:**

The online version of this article (doi:10.1186/s13063-017-2229-4) contains supplementary material, which is available to authorized users.

## Background

Statins are powerful cholesterol-lowering drugs that can reduce morbidity and mortality. On the basis of these prognostic benefits, statins have been the mainstay of treatment for atherosclerotic cardiovascular disease (CVD) [1]. Furthermore, there is mounting evidence that statins can exert beneficial cardiovascular pleiotropic effects beyond their lipid-lowering actions [2]. However, there have been persistent concerns regarding the diabetogenic property of statins. In fact, several meta-analyses have demonstrated that statin treatment is associated with a 10–12% increased risk of new-onset diabetes mellitus (NOD) [3, 4]. Notably, this risk is further increased in patients treated with intensive statin regimens [5] and in subjects with preexisting risk factors for NOD [6]. In this regard, although the implications of statin-induced diabetes on the long-term outcomes are uncertain, statin-treated patients at high risk of developing NOD should be monitored regularly for changes in plasma glucose or hemoglobin A1c [7], especially when the patients are on intensive statin treatment. However, there are no randomized clinical trials addressing this problem.

An alternative strategy to mitigate the potential risk of statin-induced diabetes might include the use of less diabetogenic statins. Although whether the diabetogenic action of statins is a class effect remains controversial, recent studies have demonstrated that pitavastatin use might be associated with neutral, or even favorable, effects on glucose metabolism [8, 9]. However, an important limitation of these studies is their short follow-up duration. Furthermore, the underlying mechanism is poorly understood, although researchers in some studies have reported the possibility that adiponectin is associated with the effect of statins on the risk of NOD [10, 11].

Thus, the purpose of the present study, the Long-term Effects of high-doSe pitavaStatin on Diabetogenicity in comparison with atorvastatin in patients with Metabolic syndrome (LESS-DM) trial, is to investigate the long-term effect of pitavastatin on glucose metabolism in comparison with atorvastatin in patients with metabolic syndrome (MetS), a group at high risk for developing NOD. In this study, we also aim to evaluate changes in adiponectin to provide insight into the relevant mechanisms leading to statin-induced diabetes. Additionally, measurements of carotid elasticity and cardiac function by using carotid ultrasound and echocardiography, respectively, will be provided as surrogate imaging endpoints to compare the potential benefits of pitavastatin versus atorvastatin on the cardiovascular system before the clinical endpoint data can be obtained [12, 13].

## Methods/design

### Study rationale and objectives

**Fig. 1:**
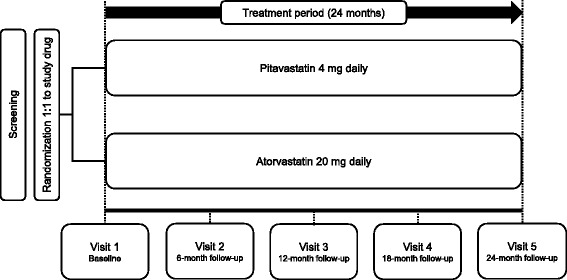
Study design of the Long-term Effects of high-doSe pitavaStatin on Diabetogenicity in comparison with atorvastatin in patients with Metabolic syndrome (LESS-DM) trial

**Fig. 2:**
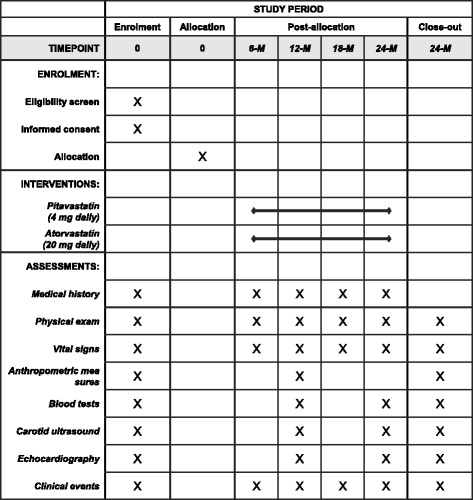
Standard Protocol Items: Recommendations for Interventional Trials (SPIRIT) figure

This trial is a prospective, multicenter, randomized, open-label, active control study. The purpose of the study is to assess the effect of pitavastatin 4 mg daily versus atorvastatin 20 mg daily on changes in various parameters reflecting glucose metabolism in patients with MetS after 24 months of treatment. The time line of the study is illustrated in Fig. 1. The Standard Protocol Items: Recommendations for Interventional Trials (SPIRIT) checklist and figure are given in Additional file 1 and Fig. 2, respectively. The trial protocol is registered with ClinicalTrials.gov (NCT02940366).

### Study participants

We will recruit from patients with MetS of both genders, aged 21 years or older, and who are candidates for statin therapy for prevention against CVD who visit outpatient clinics. These patients with MetS will be eligible only when they have no evidence of diabetes at baseline. Diabetes mellitus will be diagnosed as a random plasma glucose level ≥ 200 mg/dl along with classic symptoms of hyperglycemia, fasting plasma glucose level ≥ 126 mg/dl, 2-h postprandial plasma glucose level ≥ 200 mg/dl, and/or hemoglobin A1c ≥ 6.5%, or if a patient is taking medication for diabetes [14]. MetS will be defined according to the National Cholesterol Education Program Adult Treatment Panel III criteria [15]. The definition for anthropometric cutoff points of abdominal obesity will be based on the International Obesity Task Force criteria for the Asia-Pacific population [16]. The indication for statin therapy will be defined on the basis of the current guidelines [1]. Subjects fulfilling at least one of the following exclusion criteria will not be eligible for this study: (1) history of acute coronary syndrome or cerebrovascular disease in the past 2 months; (2) history of statin exposure within the past 1 month; (3) any anticipated change in statin regimen; (4) active malignancy; (5) cholestasis (serum total bilirubin > 3 mg/dl) or active liver disease (serum alanine transaminase > 200 IU/L); (6) chronic renal failure (serum creatinine > 2 mg/dl); (7) failure to obtain informed consent; (8) difficulty in completing follow-up; (9) concomitant use of medications affecting glucose metabolism, such as steroids and atypical antipsychotics; (10) known allergy to statins; and (11) any contraindication to statin therapy, including pregnancy/potential pregnancy, breastfeeding, cyclosporine use, myopathy, or genetic disorders.

### Randomization

After the screening phase, randomization will be performed by using a web-based computerized program hosted at the Medical Research Collaborating Center of Seoul National University Hospital. With this program, eligible participants will be randomly allocated in a 1:1 ratio to receive either pitavastatin or atorvastatin. To ensure balance of the treatment groups with respect to gender, a randomization scheme will be performed separately within the stratum of gender. Stratified randomization according to previous use of statins will also be conducted to ensure even distribution of this factor between the study groups.

### Interventions

The trial will be conducted for 24 months. After randomization, the study participants will take pitavastatin 4 mg or atorvastatin 20 mg once daily. The prescription and administration of the study medications will be performed in an open-label manner. The dose of pitavastatin was set on the basis of a previous study in which researchers reported the effect of high-dose pitavastatin on glucose homeostasis [8]. Atorvastatin was chosen as the active control because it is the most commonly used statin and has been reported to result in an increase in insulin resistance and ambient glycemia [17]. The dose of atorvastatin was selected on the basis of previous experiments comparing these two statins [18, 19]. The investigators and clinical research coordinators will assess the drug compliance of the study participants, and those with < 70% compliance will be excluded from the per-protocol analysis. In addition to medical interventions, lifestyle modifications will be encouraged as background therapy in this study. On the basis of previous studies, the patients’ smoking status, alcohol consumption, diet, and level of physical activity are considered major modifiable lifestyle factors [20, 21]. Thus, the patients will be instructed and encouraged to adhere to healthy lifestyles, including not smoking, no excessive alcohol consumption, low-risk diets, and being physically active.

### Measurements

Data on the patients’ medical history, physical examination findings, vital signs, anthropometric measures, blood biochemical tests, and imaging parameters will be collected by trained staff at our institution. Weight and height will be measured by trained nurses, and body mass index will be calculated as the weight in kilograms divided by height in meters squared. Body composition will be assessed using a multifrequency bioimpedance analyzer (InBody 720; InBody, Seoul, Korea) [22].

The primary endpoint of this study is the change in hemoglobin A1c after 24 months of statin treatment. The secondary endpoints include changes in other laboratory variables, such as insulin, C-peptide, the insulin resistance index of the homeostasis model assessment, the insulin secretory ability index of the homeostasis model assessment, lipid profiles (including total cholesterol, triglycerides, high-density lipoprotein cholesterol, and low-density lipoprotein cholesterol), adiponectin, C-reactive peptide, and troponin I, after 24 months of statin treatment. Carotid ultrasound and echocardiography will be performed at baseline and after 12 and 24 months of therapy to obtain data on carotid elasticity and cardiac function, respectively. Specifically, the carotid elasticity metrics, including strain, stiffness, and distensibility by the B-mode and speckle-tracking methods, will be measured as described previously [23]. The imaging parameters of cardiac function to be measured include the left ventricular global longitudinal strain by speckle-tracking echocardiography as well as traditional indices derived from two-dimensional, Doppler, and tissue Doppler echocardiography [24]. Additional file 2 provides a detailed description of the methods that will be used to measure the carotid ultrasound and echocardiographic parameters.

In addition, the incidences of NOD and CVD, including coronary heart disease (angina, myocardial infarction, cardiac revascularization, or coronary death), stroke (fatal or nonfatal ischemic stroke), heart failure, arrhythmia, peripheral vascular disease, and venous thromboembolism, will be assessed during the follow-up of this trial. Follow-up of these clinical events will be performed by means of in-office visits, medical record review, and telephone contact. Specifically, trained research personnel who are unaware of the study group assignments will review all medical records and will also conduct telephone interviews if needed to monitor the development of clinical events. The schedule for the assessments is summarized in Table 1.

**Table 1:** Schedule of study assessments

	V1 (Baseline)	V2 (6 months)	V3 (12 months)	V4 (18 months)	V5 (24 months)
Informed consent	√				
Medical history	√	√	√	√	√
Physical examination	√	√	√	√	√
Vital signs	√	√	√	√	√
Anthropometric measures	√		√		√
Blood tests					
Complete blood count	√				√
Routine blood chemistry	√		√		√
Lipid profile	√		√		√
Fasting plasma glucose	√		√		√
Hemoglobin A1c	√		√		√
Insulin	√		√		√
C-peptide	√		√		√
HOMA-R	√		√		√
HOMA-β	√		√		√
Adiponectin	√		√		√
High-sensitivity CRP	√				√
Cardiac troponin I	√				√
Creatine kinase	√		√		√
Carotid ultrasound					
Strain by B-mode	√		√		√
Stiffness by B-mode	√		√		√
Distensibility by B-mode	√		√		√
Strain by speckle tracking	√		√		√
Stiffness by speckle tracking	√		√		√
Echocardiography					
2D measurements	√		√		√
M-mode parameters	√		√		√
Doppler parameters	√		√		√
Tissue Doppler parameters	√		√		√
2D speckle-tracking analysis	√		√		√
Clinical events	√	√	√	√	√

*Abbreviations: HOMA-R* Insulin resistance index of homeostasis model assessment, *HOMA-β* Secretory ability of homeostasis model assessment, *CRP* C-reactive protein, *2D* Two-dimensional

### Laboratory investigations

Venipuncture will be performed on the median cubital vein of participants, and blood samples will be collected into evacuated tubes by using aseptic precautions. Urine samples will also be drawn into standard urine collection containers. All biochemical analyses, except adiponectin, will be performed in the Seoul National University Hospital Department of Laboratory Medicine, a fully accredited diagnostic laboratory. Specifically, the department of laboratory medicine of our institute is accredited by the College of American Pathologists (CAP) and has been inspected every 2 years since 2012. The proficiency testing offered by CAP has been performed for all laboratory assays, including hemoglobin A1c. With regard to the details of hemoglobin A1c measurement, venous blood samples collected in ethylenediaminetetraacetic acid bottles will be transported immediately from outpatient clinics to the department of laboratory medicine. Then, hemoglobin A1c will be measured using ion exchange high-performance liquid chromatography (VARIANT II TURBO 2.0 system; Bio-Rad Laboratories, Hercules, CA, USA). Patients will be requested to be fasting for > 10 h prior to the test, although hemoglobin A1c values reflect the average glucose level over the last several weeks. The within-laboratory coefficients of variation for the hemoglobin A1c assay are 1.26% for the normal quality control material and 0.85% for the high quality control material, respectively, representing excellent within-laboratory replication. Analysis of adiponectin will be performed at GreenCross LabCell Corp. (Yongin, Korea), an established diagnostic laboratory certified by the key domestic and international accreditation bodies, including CAP. Adiponectin will be measured by enzyme-linked immunosorbent assay (ELISA) (Human Adiponectin ELISA, BioVendor, Brno, Czech Republic; VersaMax ELISA Microplate Reader, Molecular Devices, Sunnyvale, CA, USA). Samples will be stored at −70 °C because the analysis of adiponectin will be performed as a batch, whereas other biochemical analyses will be conducted as patients present.

### Trial organization

The executive committee, composed of the study chairperson and selected members among the investigators, will govern all aspects of the LESS-DM trial. This committee will also be responsible for reviewing the final results, determining the methods of dissemination, and preparing the subsequent publications. Data coordination will be conducted at the Cardiovascular Center of Seoul National University Hospital. The data and safety monitoring board, composed of cardiologists and a biostatistician, will be responsible for making recommendations on any issue related to the safety or compliance of the patients throughout the course of the trial. This board will be able to recommend that the executive committee stop the study prematurely or modify the study protocol, although all final decisions regarding these issues will rest with the executive committee. The study sponsor, JW Pharmaceutical Corporation (Seoul, Korea), will only provide funding for this trial and will not have access to the study results until the final analyses are completed.

### Data and safety monitoring plan

The investigators and clinical research coordinators will regularly monitor all parts of the trial, including the enrollment status, medical records, case report forms, and compliance with the study protocol, during the study period. For safety evaluation, adverse events will be assessed and reported during each patient visit. All serious adverse events will be reported to the investigators and the ethics committee.

### Personal data protection and confidentiality

Confidentiality will be maintained throughout the trial by assigning all participants with a unique identification code. In order to protect participants’ anonymity, their names will not be used. The files containing the personal details of the participants along with their identifier codes will be stored separately in a locked filing cabinet and will be accessible only to the principal investigator. All data will be kept for a maximum of 3 years from the date of completion of this study and then destroyed securely.

### Sample size

The sample size was calculated by assuming an expected difference of 0.2% in hemoglobin A1c change between the groups and a population variance of 0.5%, based on previous studies [25, 26]. Thus, with a power of 95%, a two-tailed α of 5%, and a presumed dropout rate of 10%, a sample of 250 patients in each group will be sufficient to detect this difference. Accordingly, a total of 500 participants will be randomized and included in our analysis.

### Statistical analyses

The statistical analyses will be performed on both intention-to-treat and per-protocol bases. The intention-to-treat analyses will include all randomized participants who provide written informed consent, regardless of whether the study treatment is administered correctly. The per-protocol analyses will include all study participants who complete the trial with an adequate administration of the study drug (≥70% of the planned doses) and without any major protocol violations. Major protocol violations include inappropriate enrollment, the use of a prohibited concomitant medication, a violation of the dose or schedule of drug administration, or any other violation considered to be a major violation. The main analyses will be performed on an intention-to-treat basis.

The values will be expressed as the mean ± SD or median (IQR), as appropriate. Comparisons of continuous and categorical data will be made by Student’s *t* test and the chi-square test, respectively. The difference in changes between the groups will be analyzed using both Student’s *t* test and analysis of covariance to adjust for the baseline values. Two-sided *p* values < 0.05 will be considered statistically significant. All analyses will be performed using IBM SPSS version 22.0 software (IBM, Armonk, NY, USA).

## Discussion

The risk of statin-induced diabetes has been found to be increased in patients receiving an intensive statin regimen and in patients with known risk factors for NOD [5, 6]. Hence, patients at high risk for developing NOD, such as patients with MetS, should be monitored carefully, particularly when high-intensity statin therapy is used. Alternatively, selective use of statins with favorable effects on glucose metabolism may represent a feasible and safe strategy for managing these patients. Given recent reports indicating neutral or beneficial effects of pitavastatin on glucose metabolism [8, 9], pitavastatin is suggested as the preferred agent. However, the current clinical guidelines do not recommend a specific statin type in populations at high risk for developing NOD [1, 27], owing to the limited data available. Specifically, most previous studies had a fixed follow-up period of 12 weeks [7, 9], allowing only for assessment of the short-term effects of statins on glucose metabolism. More importantly, to date, there have been no clinical trials specifically designed to investigate the diabetogenic effects of statins. In this regard, a randomized trial with longer follow-up is warranted, considering that the long-term effect of statins on diabetes risk is a more clinically relevant endpoint. The LESS-DM trial will provide data on whether long-term use of pitavastatin (24 months), in comparison with atorvastatin (a representative diabetogenic statin), results in protective or deleterious effects on several parameters related to glucose metabolism in the setting of MetS. Thus, the findings of the LESS-DM trial will not only confirm or refute the previous observations regarding the favorable effects of pitavastatin on glucose metabolism but also extend knowledge about the long-term effects of intensive statin treatment on NOD.

Patients with MetS seem to represent a potential target population in which diabetogenic statin use is of particular concern, given that MetS is a constellation of interrelated risk factors for not only CVD but also diabetes [28, 29]. Because preventive strategies for CVD have been found to be clinically effective and cost-effective in general high-risk populations, the importance of integrating CVD prevention efforts has also been underscored as a cornerstone for improving the prognosis in patients with MetS [30, 31]. Although lifestyle modification is the standard first-line means of preventing CVD in these patients, this is usually not enough to reach the goal recommended in general clinical practice. Pharmacological intervention using various medications is considered an alternative and practical approach for the primary prevention of CVD. Among the currently available pharmacological agents, statins have been suggested to exert protective effects against CVD development in patients with MetS [30, 32]. Specifically, several post hoc analyses of clinical trials testing simvastatin, lovastatin, atorvastatin, or rosuvastatin for primary or secondary CVD prevention have shown that statin therapy offers significant benefits to both the subgroup of patients with MetS and the entire study population [33]. However, although the primary consideration when pharmacological interventions are contemplated for patients with MetS is the prevention of CVD, the influence of these agents on diabetes risk should also be taken into account, owing to the high risk of developing NOD in this population. In particular, there is a concern that the use of statins with diabetogenic properties may reduce the potential net benefits of statin therapy in these patients. Indeed, researchers in previous studies have reported that atorvastatin or rosuvastatin therapy is associated with a greater risk of NOD in patients with MetS than in those without [34, 35]. Thus, while the clinical benefits of statin therapy fortunately exceed the hazard of developing diabetes, even in patients with MetS [35], the magnitude of the net benefit might be attenuated with the use of more diabetogenic statin types and therefore could theoretically be enhanced further with the use of less diabetogenic ones. However, there have been no previous studies assessing whether statins with less diabetogenic properties have greater beneficial effects on the cardiovascular system, particularly in individuals at high risk for diabetes, including patients with MetS. In this context, we plan to prospectively test the hypothesis that the difference in diabetogenicity between the two statin types may contribute to differences in their impacts on cardiac and vascular functions. Among several imaging parameters reflecting cardiac and vascular functions, we plan to measure left ventricular myocardial strain and carotid artery elasticity metrics in light of previous studies demonstrating the positive effect of statin use on left ventricular function [36] and carotid artery elasticity [12]. Moreover, because left ventricular myocardial strain and carotid artery elasticity metrics have been found to have prognostic values in predicting adverse clinical outcomes [37–40], it is tempting to speculate that studies comparing effects of different statins (with different diabetogenic properties) on these imaging parameters may provide useful information for inferring the potential benefits of using less-diabetogenic statins on cardiovascular outcomes until clinical endpoint data become available. Hence, the results of the LESS-DM trial will provide intriguing insight into the long-term consequences of statin-induced diabetes. Ultimately, we consider that our findings may help clinicians in selecting the most appropriate statin type for long-term treatment of patients with MetS.

Further, adiponectin has gained increasing recognition as an important antidiabetic hormone derived from adipose tissue [10, 41, 42]. Thus, the investigation of the link between statins and adiponectin may help to elucidate the molecular mechanisms underlying statin-induced diabetes. Several previous studies, albeit conducted in small numbers of patients, have demonstrated that the effects of statins on adiponectin differ according to the type of statin used [10]. Of note, in these studies, increases in the plasma adiponectin levels were relatively consistent and almost exclusively observed in patients receiving pitavastatin [43–45], whereas the adiponectin levels were unchanged or even decreased in those treated with other statin types, including atorvastatin [17, 46–48]. These findings suggest that the adiponectin-increasing effect of pitavastatin might be causally related to its neutral or favorable impact on glucose metabolism. The LESS-DM trial results will shed light on the potential link between the statin type and diabetogenicity by assessing the continuous changes in the adiponectin levels and the relationship between these changes and several glucose metabolism parameters. Considering that hypoadiponectinemia is a main contributor to MetS [49–51], patients with MetS are an appropriate study population for assessing the adiponectin-increasing effect of statins. Additionally, some studies have suggested that adiponectin per se may be protective against arterial stiffness [52, 53] and left ventricular systolic dysfunction [54, 55]. Taken together, it can be speculated that the use of statins with positive effects on the adiponectin level might have more beneficial impacts on the vascular and cardiac functions in patients with MetS, as compared with other statins, a hypothesis that will be tested in this trial.

### Strengths and weaknesses

To date, there have been few head-to-head trials comparing the effects of different statins on glucose metabolism. Therefore, our study has an advantage in this respect because we will directly compare the effect of pitavastatin with that of atorvastatin, one of the most widely used statins clinically, on glucose metabolism and NOD. Another strength of this study is the provision of information regarding the impact of statins on adiponectin, which may give insight into the potential mechanisms underlying the difference in diabetogenic properties according to the statin type. This study also has an advantage in that it will include analyses of the effects of statin therapy on imaging surrogate markers, such as carotid elasticity metrics and echocardiographic parameters. On one hand, the results of these analyses will address the question whether the use of less-diabetogenic statin types has more beneficial effects on vascular and cardiac functions in patients at high risk for diabetes. On the other hand, the major limitation of our study is that the sample size is relatively small to detect differences in clinical outcomes, such as NOD or cardiovascular events, between the groups. To mitigate this limitation, we plan to use imaging surrogate endpoints, which may allow for a smaller sample size and a shorter study duration than clinical endpoints and thereby enable the assessment of the potential benefits of pitavastatin on cardiovascular outcomes. However, we should acknowledge that this approach based on imaging surrogate indicators cannot be fully applied to NOD. Another limitation is a lack of power to perform reliable subgroup analyses based on relevant variables. For example, given that age is a major risk factor for diabetes, subgroup analyses looking at narrower age ranges will provide additional valuable information on potential age group differences in the risk of statin-induced diabetes. Large clinical trials need to be conducted to determine which age group is the most susceptible for developing statin-induced diabetes or most benefits from the use of less-diabetogenic statins. With regard to gender, although there are no direct data on gender differences in the association between statin use and NOD, some previous studies suggested that diabetogenic potential of statins might be gender-specific by demonstrating that the baseline level of adiponectin and the effect of statins on adiponectin could be different between males and females [56, 57]. Further trials with adequate numbers of males and females are required to be able to answer the diabetogenic potential of statins based on gender differences. We should also acknowledge that the influence of previous statin use on the study endpoints cannot be completely excluded, even though we will exclude patients with a history of statin exposure within 1 month before enrollment and thus will perform stratified randomization according to the previous use of statins. Finally, patients receiving some antihypertensive agents with potential to affect glucose metabolism, such as thiazide-type diuretics, β-blockers, angiotensin-converting enzyme inhibitors, and angiotensin receptor blockers, which may be important covariates, can be included in our study [58]. If these covariates are not evenly distributed between study groups despite randomization, we will perform adjusted analysis in this randomized controlled trial to correct for the imbalances in covariates.

## Conclusions

**Fig. 3:**
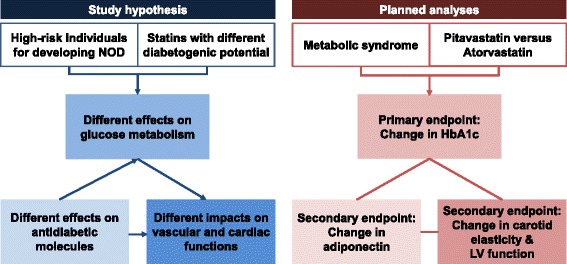
Simplified schematic diagram demonstrating the study hypothesis and associated analyses. We hypothesize that pitavastatin may have a more favorable influence on glucose metabolism than atorvastatin, potentially mediated by its unique adiponectin-increasing effect. The possible antidiabetic property of pitavastatin may contribute to improvements in vascular and cardiac functions in patients with metabolic syndrome, a high-risk group for developing new-onset diabetes mellitus. The Long-term Effects of high-doSe pitavaStatin on Diabetogenicity in comparison with atorvastatin in patients with Metabolic syndrome (LESS-DM) trial is specifically designed to explore this hypothesis by measuring the change in hemoglobin A1c (primary endpoint); changes in biochemical markers, including adiponectin; and changes in imaging markers, including carotid elasticity metrics and left ventricular function parameters, after statin therapy (secondary endpoints). *NOD* New-onset diabetes mellitus, *HbA1c* Hemoglobin A1c, *LV* Left ventricle

In the LESS-DM trial, we will investigate the impact of high-dose pitavastatin on diabetogenicity in comparison with that of atorvastatin in patients with MetS. In this study, we hypothesize that (1) pitavastatin will have beneficial effects on the levels of circulating biomarkers reflecting glucose metabolism; (2) the differences in diabetogenic properties among the statin types may stem from differences in their action on adiponectin; and (3) statins with favorable effects on glucose metabolism will also relate to improvements in vascular and cardiac functions (Fig. 3). The results of the LESS-DM trial will serve as an important step toward a larger clinical outcome trial testing the statin type-dependent effects on diabetogenicity and the consequences thereof in patients with MetS. Moreover, this trial will provide a cornerstone for statin therapy tailored to specific subgroups at high risk for NOD, including patients with MetS.

## Trial status

This trial is in the recruitment stage (protocol version 2.0, protocol approval date 7 November 2016, recruitment of participants start date 2 December 2016, and expected recruitment completion date 30 November 2019).

## Additional files

Additional file 1: SPIRIT 2013 checklist: recommended items to address in a clinical trial protocol and related documents. (DOCX 48 kb)

Additional file 2: Supplementary methods for details of imaging protocols. (DOCX 26 kb)

## Abbreviations

CAP: College of American Pathologists
CRP: C-reactive protein
CVD: Cardiovascular disease
2D: Two-dimensional
ELISA: Enzyme-linked immunosorbent assay
HOMA-β: Secretory ability of homeostasis model assessment
HOMA-R: Insulin resistance index of homeostasis model assessment
LESS-DM: Long-term Effects of high-doSe pitavaStatin on Diabetogenicity in comparison with atorvastatin in patients with Metabolic syndrome trial
LV: Left ventricle
MetS: Metabolic syndrome
NOD: New-onset diabetes mellitus
SPIRIT: Standard Protocol Items: Recommendations for Interventional Trials
