# Supplementary material for: Long-term Effects of high-doSe pitavaStatin on Diabetogenicity in comparison with atorvastatin in patients with Metabolic syndrome (LESS-DM): study protocol for a randomized controlled trial
Source: Trials. 2017 Oct 27;18:501. doi: 10.1186/s13063-017-2229-4 (PMC5659042; doi:10.1186/s13063-017-2229-4)
Supplement: Supplementary file 2 — Supplementary methods for details of imaging protocols. (DOCX 26 kb) [file 13063_2017_2229_MOESM2_ESM.docx]

**Carotid ultrasound**

Ultrasound images will be acquired with a GE Vivid E9 system (GE Healthcare) and a 7.5-MHz linear 2-dimensional (2D) array transducer, following a standardized protocol.^1^ The distal left common coronary arteries (CCA) will be imaged 10-mm inferior to the carotid bulb in transverse and longitudinal sections with electrocardiography-gating over at least 3 cardiac cycles. Images will be transferred to a workstation equipped with 2D strain software (EchoPAC version 113; GE Healthcare). Conventional carotid artery elasticity metrics and intima-media thickness (IMT) will be assessed using B-mode ultrasound as previously described.^2^ Briefly, systolic (Ds) and diastolic diameters (Dd) will be measured and averaged over 3 cardiac cycles. Systolic (SBP) and diastolic BP (DBP) will be measured before and after the carotid ultrasound examination and then averaged. Carotid IMT will be defined as the distance between the leading edges of the first and second echogenic lines, representing the lumen-intimal interface and the upper layer of the adventitia, respectively. Elasticity variables will be calculated as follows: strain by B-mode (%) = (Ds-Dd)/Dd, classic stiffness index (β1) = ln(SBP/DBP)/strain by B-mode ultrasound, and distensibility = 1/[ln(SBP/DBP)/strain by B-mode ultrasound × IMT]. We will also perform carotid arterial strain (CAS) analysis using speckle-tracking as previously described.^3-6^ Briefly, all the regions of interest (ROI) will be placed to cover the cross-sectional area of the CCA wall. The software will automatically detect frame-to-frame movement of each speckle on the CCA wall during the cardiac cycle. The CCA wall will be equally divided into 6 segments and each segment will be analyzed individually. From each time-strain curve for all 6 segments, the global circumferential peak systolic strain (%) and early and late systolic strain rate (strain per time unit, s^-1^) will be determined automatically. In addition, speckle-tracking-derived stiffness index (β_2_) will be calculated as follows: β_2_ = ln(SBP/DBP)/strain by speckle-tracking.^7, 8^

**Echocardiography**

We will perform transthoracic echocardiography with a standard protocol in all patients before and after 3 months of pitavastatin therapy. Images will be acquired using a GE Vivid E9 ultrasound system (GE Healthcare) with a standard M5S transducer. A workstation with dedicated software (EchoPAC version 113) will be used for offline analysis. Speckle-tracking echocardiography will be used to measure left ventricular global longitudinal strain as previously described.^9^ In brief, we will acquire 2D images from 3 standard apical views (apical 4-chamber, 2-chamber, and 3-chamber views). The left ventricular endocardial border will be manually traced in the end-systolic phase with a caution not to include the pericardium. Subsequently, EchoPAC software will automatically trace the endocardium in the other frames and determine 6 segments in each view. The software will semi-automatically define the ROI between the endocardial and epicardial borders and track the frame-to-frame movement of left ventricular segments. Readings will be obtained by averaging 6 segments in each view and left ventricular global longitudinal strain will be determined as the averaged peak strain of all 18 segments.

**References**

1. Godia EC, Madhok R, Pittman J, Trocio S, Ramas R, Cabral D, et al. Carotid artery distensibility: a reliability study. J Ultrasound Med 2007;26(9):1157-65.

2. Ratchford EV, Gutierrez J, Lorenzo D, McClendon MS, Della-Morte D, DeRosa JT, et al. Short-term effect of atorvastatin on carotid artery elasticity: a pilot study. Stroke 2011;42(12):3460-4.

3. Bjallmark A, Lind B, Peolsson M, Shahgaldi K, Brodin LA, Nowak J. Ultrasonographic strain imaging is superior to conventional non-invasive measures of vascular stiffness in the detection of age-dependent differences in the mechanical properties of the common carotid artery. Eur J Echocardiogr 2010;11(7):630-6.

4. Kim SA, Park SM, Kim MN, Kim YH, Cho DH, Ahn CM, et al. The relationship between mechanical properties of carotid artery and coronary artery disease. Eur Heart J Cardiovasc Imaging 2012;13(7):568-73.

5. Saito M, Okayama H, Inoue K, Yoshii T, Hiasa G, Sumimoto T, et al. Carotid arterial circumferential strain by two-dimensional speckle tracking: a novel parameter of arterial elasticity. Hypertens Res 2012;35(9):897-902.

6. Yang EY, Dokainish H, Virani SS, Misra A, Pritchett AM, Lakkis N, et al. Segmental analysis of carotid arterial strain using speckle-tracking. J Am Soc Echocardiogr 2011;24(11):1276-1284 e5.

7. Teixeira R, Vieira MJ, Goncalves A, Cardim N, Goncalves L. Ultrasonographic vascular mechanics to assess arterial stiffness: a review. Eur Heart J Cardiovasc Imaging 2016;17(3):233-46.

8. Oishi Y, Miyoshi H, Iuchi A, Nagase N, Ara N, Oki T. Vascular aging of common carotid artery and abdominal aorta in clinically normal individuals and preclinical patients with cardiovascular risk factors: diagnostic value of two-dimensional speckle-tracking echocardiography. Heart and vessels 2013;28(2):222-228.

9. Lee SP, Kim HK, Kim YJ, Oh S, Sohn DW. Association of myocardial angiogenesis with structural and functional ventricular remodeling in aortic stenosis patients with normal ejection fraction. J Cardiovasc Ultrasound 2014;22(2):72-9.
